# A Method of Managing Waste Oak Flour as a Biocomponent for Obtaining Composites Based on Modified Soybean Oil

**DOI:** 10.3390/ma15217737

**Published:** 2022-11-03

**Authors:** Anna Sienkiewicz, Piotr Czub

**Affiliations:** Department of Chemistry and Technology of Polymers, Faculty of Chemical Engineering and Technology, Cracow University of Technology, Warszawska Str. 24, 31-155 Kraków, Poland

**Keywords:** wood flour, biocomposite, epoxy resin, epoxy fusion process

## Abstract

The aim of the present research was the development of a management method for wood-processing waste that was obtained during the production of parquet flooring. Currently mostly useless, such waste mainly ends up in landfills. The oak waste flour was used as a reinforcement material for epoxy biocomposites based on the polyaddition product of epoxidized soybean oil and bisphenol-A (ESBO_BPA). The biofiller was subjected to mercerization, acetylation, and diisocyanate modification to increase the typically poor compatibility between the highly hydrophilic wood fibers and the hydrophobic polymer matrix. Among the analyzed epoxy biocomposites, which contained about 60% raw materials of natural origin, it was found that the best mechanical properties were recorded for cured samples of the ESBO_BPA composition filled with 5 wt % of oak flour mercerized using a 5% solution of NaOH. It was also proven that a higher concentration of alkali deteriorated the mechanical-strengthening properties of the wood filler. The acetylation of the biofiller independently in the best elimination of hydroxyl groups from its structure also removed irregular strips and smoothed its surface. This resulted in a poorer wettability of the oak flour surface by the polymer and consequently an easier pullout of the filler from the polymer matrix and worse mechanical properties of the wood/epoxy composite. To the best of the authors’ knowledge, the present research was the first to examine the possibility of the application of parquet flooring post-production wood flour in biomaterials based on a polyaddition product of epoxidized soybean oil and bisphenol-A.

## 1. Introduction

Due to the continual increase in the scale of production of polymeric materials and the accompanying environmental and economic problems, scientists are paying more attention to the use of renewable materials in the synthesis of plastics [1,2]. The constant demand for fossil raw materials for the production of polymers leads to their depletion, and finally, their smaller resources may cause an increase in the prices of final products. Another important issue that is motivating the search for novel raw materials is generally a low degree of degradability and tendency of forming micro- and nanoplastics, leading to the problem of the rising scale of pollution and climate change issues [3,4,5]. Therefore, raw materials of natural origin may constitute competition for raw materials of petrochemical origin and hopefully contribute to lowering the costs of plastics as well as their harmful impacts on the environment in the future [6,7]. 

Among the renewable raw materials used in the synthesis of polymeric materials, one can distinguish among others vegetable oils [8,9], polysaccharides [10,11], starch [12,13], and wood [14,15]. These raw materials of natural origin are used either in the process of synthesis [16] or as fillers for green plastic composites [17,18]. Even though nowadays numerous published studies have demonstrated the possibility of using renewable raw materials to obtain polymer-based materials, the final products are often characterized by worse properties compared to plastics based on petrochemical raw materials, hence there is a constant need for further research.

Epoxy resins are an example of polymeric materials that are under constant research in an effort to improve the ecological aspects of their synthesis and application. Despite several advantages, cross-linked epoxy resins are brittle and rigid materials that are characterized by a relatively low elongation at break as well as a low impact strength [19]. Hence, to improve the functional properties of epoxy resins, various types of modifications are used [20,21]. Nevertheless, due to the generally very good properties of epoxy resins, these materials are widely used as construction materials and are successfully used in the composite industry, among others. Epoxy composites are effectively applied in the production of automotive components such as radiator supports, bumper bars, fenders, or roof panels [22,23]. Moreover, epoxy materials that are reinforced with synthetic fibers are being increasingly used in aviation structures [24,25,26] due to their better properties such as a good fatigue strength, a high stiffness, and a low density. 

One of the methods that is commonly used for the modification of epoxy resins in the improvement of their properties is the reinforcement of the polymer matrix with various fillers. Generally, among the fillers intended for the strengthening of epoxy resins, one can distinguish between inorganic, organic, synthetic, and natural fillers. The most popular inorganic fillers include chalk [27,28], kaolin [29], silica [30,31], mica [32,33], quartz, various types of metal oxides, and metallic powders [34]. These fillers are most often in powder form; the main advantages of their application in polymeric composites are a reduction in the cost of the finished product, improvements in thermal stability, and reductions in processing shrinkage [35]. Another method of improving the mechanical and thermal properties of polymeric materials is modification with fibers. Among synthetic fibers, one can distinguish those composed of glass [36], quartz, boron [37], silicate [38], carbon, graphite [39], and whiskers. Nowadays, due to the growing interest both in biodegradable recyclable materials and in finding novel ways to manage waste materials, the use of fillers of natural origin is becoming more widely accepted in the plastics industry. These additives can be in the form of powders, flour, or fibers. The unquestionable advantage of the application of natural reinforcements over synthetic ones is that they are harmless to the environment and are easier to modify and process. The incorporation of natural fibers into epoxy resins typically results in an improvement in the mechanical and thermal properties. Kumar et al. [40] found that the application of raw wood particles obtained during the cutting of wood that were modified with sodium hydroxide led to a better thermal stability at a higher temperature of the composite material based on AW 106 epoxy resin. The observed phenomenon was explained by the increased crystalline area in the filler’s molecular structure and the removal of lower-molecular-weight natural polymers. Jain and Gupta [41] stated that the reinforcement of an epoxy composite with 33 wt % of sal and teak wood floor in a 1:1 ratio resulted in improved mechanical properties and water intake of the obtained material. Dinesh et al. [42] presented the influence of two different wood dust fillers in jute-fiber-based epoxy composites. It was found that the applied wood fillers differed in their degree of adhesion with the polymer matrix. Therefore, for padauk wood-dust-based jute epoxy composites, higher values of tensile, flexural strength, compressive strength, hardness, and energy absorbed during impact were recorded than for rosewood-dust-based jute epoxy composites. Simultaneously, the thermal stability of the rosewood dust filler and its composites was higher than that of the padauk wood dust and epoxy composites reinforced with this filler. Nevertheless, the problem related to the introduction of natural fibers into composites is the relatively low adhesion between the fibers and the polymer material, as well as fairly high moisture sorption by the filler [35]. Based on previous research [9,43,44], it was found that the use of natural fibers (palm, ramie, coconut, bamboo, hemp, flax, sisal, jute, or kenaf) may be an alternative solution for the reinforcement of composite materials with synthetic fibers.

The research presented within this manuscript concentrated on the examination of the effect of the application of waste oak flour from parquet flooring processing in wood/polymer composites based on high-molecular-mass epoxy resin obtained via the epoxy fusion product of epoxidized soybean oil and bisphenol-A. The presented process of the synthesis of biocomposites included the synthesis of the biobased epoxy polymeric matrix and the preparation of the biofiller. In our opinion, research on such materials brings benefits in the form of obtaining interesting materials with a potential application, as well as in the functional management of wood-processing waste, which, unfortunately, mainly ends up in landfills.

## 2. Materials and Methods

Materials. We used epoxidized soybean oil (ESBO, Ergoplast EG, Boryszew, Warsaw, Poland, EV = 0.363 mol/100 g; with an average of 3.52 epoxide groups and 0.03 hydroxyl groups per 1 oil molecule), bisphenol A (BPA, Cartagena Lexan, Murcia, Spain, 99.93%), LiCl (Merck, Kenilworth, NJ, USA, pure), and oak wood waste (provided by FHU Parkiety Smolik, Lanckorona, Poland, and obtained during the production of parquet flooring).

### 2.1. Epoxy Fusion

The process was performed using epoxidized soybean oil, bisphenol A, and LiCl (in an amount of 0.002 mol per 1 mol of OH groups). The process was conducted according to a previously described procedure [45] and resulted in a polyaddition product—ESBO_BPA—which was subjected to an analysis of its contents of epoxy and hydroxy groups using both titration and FT-IR methods.

### 2.2. Wood Waste Preparation

A laboratory shaker for sieve analysis was used for the separation of waste received from the producer into homogeneous fractions, resulting in the obtainment of seven different medium dimensions of wood flour particles: >0.2 mm, 0.2–0.16 mm, 0.16–0.1 mm, 0.1–0.076 mm, 0.076–0.056 mm, 0.056–0.04 mm, and <0.04 mm. For the studies described within this manuscript, a fraction with a size <0.04 mm was used. Before its further application as a component of epoxy composites, the wood flour was predried at a temperature of 80 °C for 48 h and subjected to mercerization (1) and acetylation (2).

### 2.3. Synthesis of Composites 

The composites were based on epoxidized soybean oil filled with modified wood waste. The proper amounts of the polyaddition product (ESBO_BPA) and the wood waste (in the amount of 2 and 5 wt %) was thoroughly mixed using a mechanical mixer (700 rpm) for 30 min, followed by additional mechanical mixing (5 min, 1000 rpm) of a weighed amount of 4,4’-methylene diphenyl diisocyanate (MDI) and a deaerating agent (BYK A530, 1 wt % concerning the total weight of the composition). Next, the prepared composition was deaerated for 3 min at a pressure of 0.8 MPa. Finally, it was transferred into Teflon molds to obtain samples for mechanical tests using paddles, beams, and rollers. The curing process was conducted at room temperature for 24 h followed by an additional crosslinking process carried out at 80 °C for 24 h.

### 2.4. The Mechanical Properties 

The tensile strength, elongation at break, modulus elasticity, flexural strength, elasticity flexural modulus, deflection, compressive strength, and compression set were tested on a Zwick 1445 apparatus (Wroclaw, Poland) using the PN-EN ISO 527-1:2012, PN-EN ISO 178:2011, and PN-EN ISO 604:2006 standards. The PN-EN ISO 868:2005 standard was applied to determine the value of hardness in the Shore A scale using an InSize apparatus and the PN-EN ISO 179-2:2001 standard was applied to test the impact strength without notches via the Charpy method using a ZORN PSW 4J digital apparatus (ZORN INSTRUMENTS GmbH & Co. KG, Stendal, Germany).

### 2.5. Spectroscopic Measurements

A transmittance FT-IR spectroscopy analysis was conducted using an FT-IR PerkinElmer spectrophotometer (SPECTRUM 65 FT-IR, PerkinElmer, Seer Green, UK) with an ATR adapter. The analyses were carried out at room temperature. Spectra were recorded for a wavenumber in the range of 4000–600 cm^−1^. The obtained spectra are presented using the dependence of transmittance T (%) and wavenumber v (cm^−1^).

### 2.6. Morphological Analysis 

A morphological analysis of the obtained materials was conducted using a JEOL JSM-6010LA scanning electron microscope (Tokyo, Japan) at a 5 kV acceleration. SEM micrographs were recorded of the impact-fractured surfaces of the cured compositions. The approximate dimensions of the samples were 2 × 10 mm. The surface of the samples was coated with a thin film of gold.

## 3. Results

### 3.1. Synthesis of the Biocomposites Based on Modified Soybean Oil Filled with Waste Oak Flour

#### 3.1.1. Synthesis of the Polymeric Matrix

**Figure 1:**
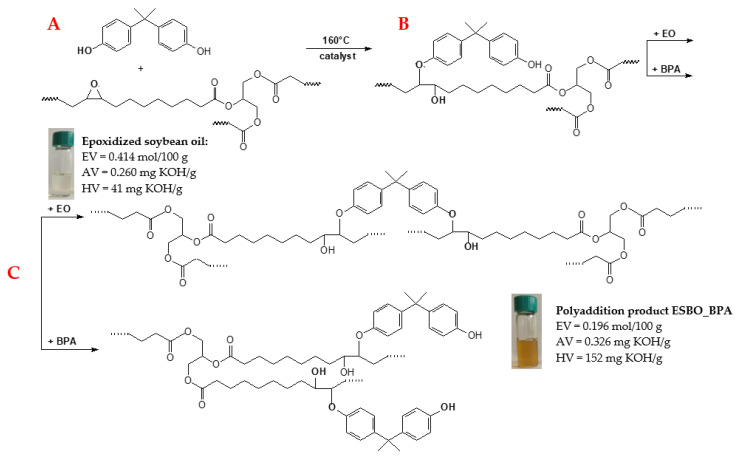
The epoxy fusion process for epoxidized soybean oil and bisphenol-A: (**A**) reaction between molecules of epoxidized oil and bisphenol; (**B**) linear oligomer formation; (**C**) subsequent reactions leading to the formation of branched oligomers.

The process of the synthesis of the biocomposites based on modified soybean oil filled with waste oak flour was divided into two stages: (1) the synthesis of the biobased polymeric matrix and (2) the preparation of the biofiller. In the first stage of the experiment, the epoxy fusion process was performed. A commercial epoxidized soybean oil (ESBO) was used as a raw material for the synthesis of a high-molecular-weight epoxy resin, which was conducted via a polyaddition reaction with bisphenol-A. The grammage of the reactants required for the synthesis was selected based on the dependence while assuming that the obtained macromolecular product would contain the epoxy groups in an amount of 0.100 mol/100 g. The polyaddition reaction was carried out at 160 °C under an inert gas atmosphere and in the presence of lithium chloride as a catalyst. As indicated in our previously published paper [46], the use of LiCl was conditioned by the fact that this catalyst directly affected the properties of the obtained product. As a result of the performed reaction, it was possible to obtain the product in a liquid form with a high viscosity. Moreover, LiCl prevented gelling of the reaction mixture by inhibiting the branching reaction. The reaction was carried out for 8 h. The final product was in the form of a viscous liquid with a brown-orange color and an optimal viscosity, ensuring a relatively easy dispersion of the biofiller. The obtained product was characterized via the determination of the content of epoxy and hydroxyl values (Figure 1). 

Based on the determined content of the end groups in the polyaddition product, we concluded that some of the epoxy groups contained in the ESBO formed BPA particles. As a result of this reaction, the epoxy rings were opened, which contributed to the increase in the content of the hydroxyl groups in the final product. 

#### 3.1.2. Preparation of the Biofiller

In the next stage of the synthesis of the oak waste/ESBO_BPA biocomposites, the wood flour was prepared. Before the final application, the waste wood flour was fractionated and the fraction with a size <0.04 mm was used. Next, it was subjected to various chemical modifications to increase the compatibility between the hydrophobic polymer matrix and the hydrophilic biofiller. The wood particles, not the favorable hydrophilic chemical structure, additionally contained the residues of waxy substances. Such characteristics of the natural filler, unfortunately, resulted in ineffective bonding between the components of the prepared composite. Hence, the chemical modification of wood flour was extremally important for its further application as a biofiller for the polymeric matrix. Moreover, the hydroxyl groups in the unmodified wood were not readily accessible for most chemical interactions until they were pretreated with sodium hydroxide. It was proven that this method eliminated not only impurities of the natural filler, but also a large amount of hemicellulose, lignin, and waxy substances [47]. Moreover, treating the surface with alkali resulted in a swelling of the cellulose, leading to the relaxation of the natural crystalline structure [48]. 

**Figure 2:**
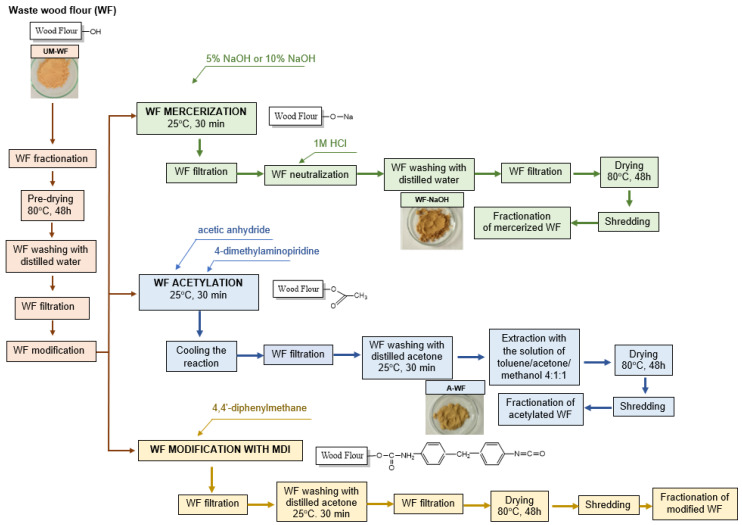
Scheme of the modification of waste oak flour.

Before the mercerization, the oak waste flour was washed with hot distilled water to initially rinse off the surface of the biofiller of the contaminations. The proper mercerization was carried out for 30 min using 5% and 10% sodium hydroxide solutions. Next, the wood waste was neutralized, rinsed with distilled water, and filtered in a Büchner funnel. Finally, the modified oak waste was dried at 80 °C for 48 h, ground, and refractionated using a laboratory shaker for a sieve analysis. During the mercerization process, the hydroxyl groups of the cellulose contained in the oak wood waste were converted into ONa groups (Figure 2), which resulted in the expansion of the dimensions of the molecules, consequently resulting in the conversion of cellulose I to a new crystalline structure of cellulose II [49]. Here, it is worth mentioning that such transformation closely depended on the concentration of the solution and treatment time. 

The raw oak filler was also subjected to modification via acetylation. This process performed on the wood material allowed for the elimination of the hydroxyl groups contained in its structure and accordingly led to better adhesion between the WF and the polymer matrix. The acetylation of the wood waste was carried out with acetic anhydride in the presence of 4-dimethylaminopyridine as a catalyst. The reaction was carried out at 120 °C for 2.5 h. Then, the reaction mixture was cooled and filtered in a Büchner funnel. The resulting waste was washed with acetone and subjected to Soxhlet extraction. The extraction was performed within 9 cycles in the presence of a toluene:acetone:methanol mixture (4:1:1 by volume); the entire process took 2 h. The obtained modified wood waste was dried at a temperature of 80 °C for 48 h. Then, the modified wood flour was ground and sieved again to finally obtain the acetylated wood flour with a size <0.04 mm.

Since we originally planned to use MDI-based polyisocyanate (DESMODUR VL, Covestro AG, Leverkusen, Germany) as a hardener for the obtained final biocomposites, we also performed the modification of the wood filler with a 4,4’-diphenylmethane diisocyanate (MDI). The suspension containing oak wood waste and MDI was mechanically stirred for 1 h. Then, the wood waste was filtered off in a Büchner funnel. Next, the drained WF was washed/filtered twice with acetone for 30 min cycles. Finally, the modified wood flour (D-WF) was dried for 24 h at 80 °C, ground, and sieved to obtain a fraction <0.04 mm. Unfortunately, the trials performed to obtain a cured composition containing isocyanate-modified wood flour failed. The obtained samples were characterized by an unsatisfactory appearance. Regrettably, we obtained porous—instead of solid—material containing numerous air bubbles. Such a situation most likely was related to the faster rate of the undesirable reaction of the -NCO groups with moisture from the air than with the hydroxyl groups from the ESBO_BPA polyaddition product. Additionally, it is worth highlighting here that such an effect was doubled because the -NCO groups were included both in the modified oak filler and in the used hardener. Therefore, no further tests including D-WF were carried out.

##### Results of the FTIR Analysis of the Wood Filler

**Figure 3:**
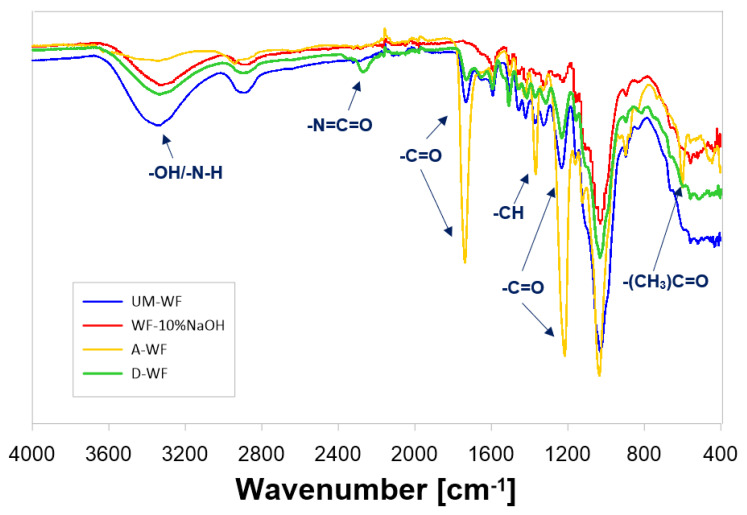
FT-IR spectrum of unmodified and chemically modified waste oak flour (UM-WF—unmodified wood flour; WF-10%NaOH—wood flour mercerized using 10% solution of NaOH; A-WF—acetylated wood flour; D-WF—wood flour modified with MDI).

The oak waste, both modified and unmodified, was analyzed using spectroscopy measurements and by recording SEM microphotographs. The spectrum (Figure 3, Table 1) presented the post-modification changes in the chemical structure of the oak wood flour. One of the most favorable post-modification changes in the structure of wood filler was noticed in the section that is typical for hydroxyl group vibrations. Such signals were observed in the range v = 3607–3080 cm^−1^. The intensity of this band, which is typical for stretching vibrations of the hydroxyl groups, was the highest in the case of the unmodified wood flour (UM-WF) compared with the visible reduction in all spectra registered for the modified and analyzed oak flour waste. Simultaneously, the relatively lowest intensity within the discussed section was registered for the spectrum of A-WF. Additionally, when comparing the spectrum of the wood flour mercerized with a 5% and 10% solution of NaOH (WF-5%NaOH and WF-10%NaOH, respectively), the influence of the concentration of the alkali solution used for the modification was observed in the different intensity of the band in the range of v = 3607–3080 cm^−1^. Surprisingly, the reduction in the content of hydroxyl groups in the wood waste modified with a 5% solution of NaOH was characterized by a lower intensity than in the case of the WF modified with the 10% solution of NaOH. Next, the transmittance band registered in the wavenumber range v = 2980–2822 cm^−1^ was related to the presence of stretching vibrations that originated from methyl and methylene groups. A significant difference noticed between the registered spectra was in the appearance of the vibration at the wavenumber at v = 2271 cm^−1^, which corresponded to the interaction of free -NCO groups [50]. The signal at v ≈ 1728 cm^−1^ was most likely related to the presence of carbonyl stretching bonds in hemicelluloses and lignin. Small signals at the wavenumber v ≈ 1590 cm^−1^ could be attributed to the -C=C- skeletal vibrations in the aromatic rings of the lignin structure. In the spectrum of A-WF, the signal at v ≈ 1365 cm^−1^ may have been related to deformation -CH vibrations in cellulose and hemicelluloses. It is also worth mentioning that in this section, the change in the intensity of the vibration may have designated the overlapping of bands corresponding to -CH groups with a signal, which is typical for the acetyl group (stretching -C-CH_3_ vibrations and bending -C-H vibrations), and possibly also occurred in this range. The band in the range v = 1297–1188 cm^−1^ most likely corresponded to the -C-O stretching vibration from lignin, while the intense band at v = 1178–883 cm^−1^ was correlated with the interaction of bonds of lignin and cellulose molecules. Based on a previous publication [50], the intensity of that band may have been affected by C-O-C stretching vibrations in pyranose rings and -C-O vibrations in aliphatic groups. The signal at v ≈ 600 cm^−1^ that can be noticed on the recorded A-WF spectrum also deserved attention; it corresponded to the deformation vibrations of the acetyl groups introduced by the conducted modification of the oak flour. 

##### Results of the Morphological Analysis of the Wood Flour

**Figure 4:**
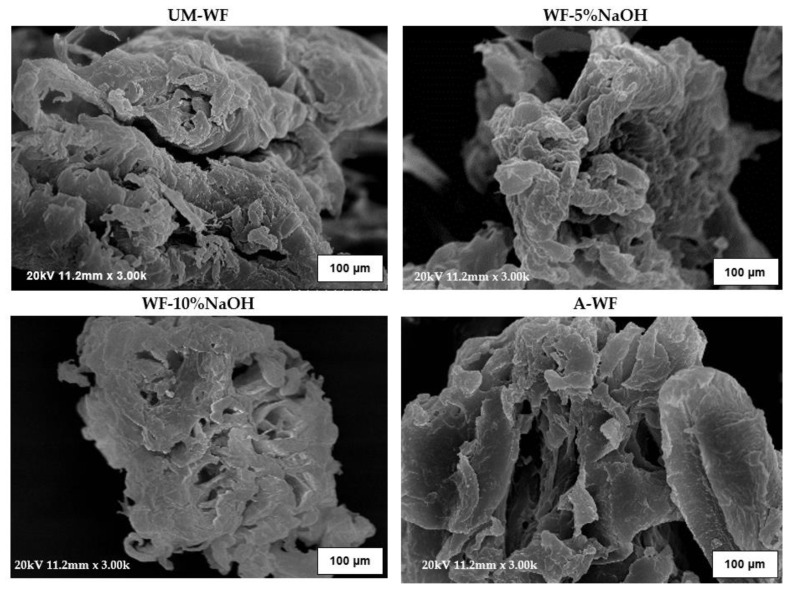
SEM micrographs of unmodified and modified waste oak wood flour.

The performed chemical interference with the biofiller had a great impact both on its chemical structure, which was visible in the FT-IR spectrum, and also on changes in the morphological appearance of the wood particles. In general, the surface of oak flour presented in the SEM microphotographs (Figure 4) was rough with numerous irregular strips that probably resulted from the wood processing performed earlier. 

Here, it is worth mentioning that when comparing all of the analyzed wood samples, the mercerized WF was characterized by the most irregular surface. This was probably due to the most effective removal of the residues of pectin, lignin, hemicellulose, waxy substances, and impurities that occurred on the wood surface. In light of this, the mentioned irregular scraps were not visible on the microphotographs of the acetylated wood filler. Additionally, due to the modification, an enlargement of wood particles was also observed that was the most visible in the case of the mercerized samples. For the unmodified wood samples, most of the wood particles were about 23–39 µm, with single larger particles—such as one of about 91 µm in the case of WF-5%NaOH—and mean particle sizes that varied from 46 to 68 µm, with some that were about 102 µm. Such a microscopic observation corresponded to swelling of the filler during the modification reaction, which was related to the response of cellulose to the alkali treatment. During mercerization, the natural crystalline structure of cellulose relaxes [49]. Such a transformation of lignocellulosic materials is especially desirable when considering further modifications. Throughout the mercerization, the decrystallization of cellulose was observed, leading to an increase in the average content of the amorphous regions, which were more prone to chemical penetration than the crystalline regions [47]. 

#### 3.1.3. The Preparation of Biocomposites

Finally, wood flour in the form of (1) unmodified oak wood waste, (2) mercerized oak wood waste, and (3) acetylated oak wood waste was introduced into the ESBO_BPA polyaddition product prepared in the first stage of the experiment in an amount of 2 or 5 wt % based on the total weight of the composition. Then, the entire mixture was thoroughly stirred mechanically for 30 min, followed by the addition of BYK A530 defoamer (1 wt % based on the total weight of the prepared composition) to facilitate the removal of air bubbles formed during mixing. Finally, 4,4’-diphenylmethane diisocyanate (MDI) was added in an amount based on the content of hydroxyl groups in the polyaddition product and poured into Teflon molds. Then, after the materials had hardened (48 h), the specimens for mechanical tests were taken out of the molds and subjected to crosslinking at a temperature of 80 °C (24 h). The following compositions were obtained: reference sample ESBO_BPA, ESBO_BPA composition filled with 2 wt % of unmodified wood flour (ESBO_BPA_2%WF), ESBO_BPA composition filled with 5 wt % of unmodified wood flour (ESBO_BPA_5%WF), ESBO_BPA composition filled with 2 wt % of wood flour mercerized using a 5% solution of NaOH (ESBO_BPA_2%WF-5%NaOH), ESBO_BPA composition filled with 5 wt % of wood flour mercerized using a 5% solution of NaOH (ESBO_BPA_5%WF-5%NaOH), ESBO_BPA composition filled with 2 wt % of wood flour mercerized using a 10% solution of NaOH (ESBO_BPA_2%WF-10%NaOH), ESBO_BPA composition filled with 5 wt % of wood flour mercerized using a 10% solution of NaOH (ESBO_BPA_5%WF-10%NaOH), ESBO_BPA composition filled with 2 wt % of acetylated wood flour (ESBO_BPA_2%A-WF), and ESBO_BPA composition filled with 5 wt % of acetylated wood flour (ESBO_BPA_5%A-WF). Depending on the applied amount of wood flour, the obtained wood/polymer composites were based on 57% or 60% raw materials of natural origin. 

### 3.2. Mechanical Properties of ESBO_BPA/Wood Flour Composites

**Figure 5:**
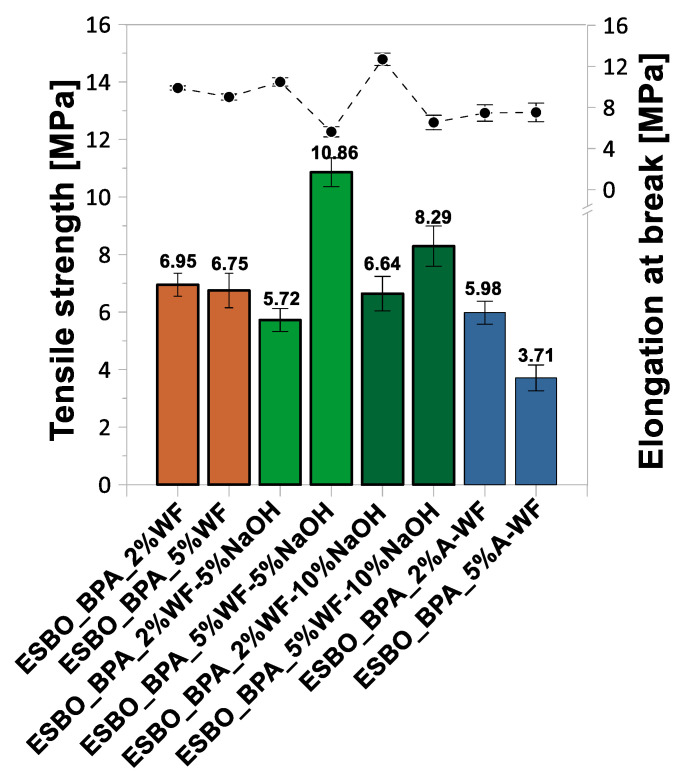
Tensile strength and elongation at break of compositions based on ESBO_BPA filled with waste oak flour.

**Figure 6:**
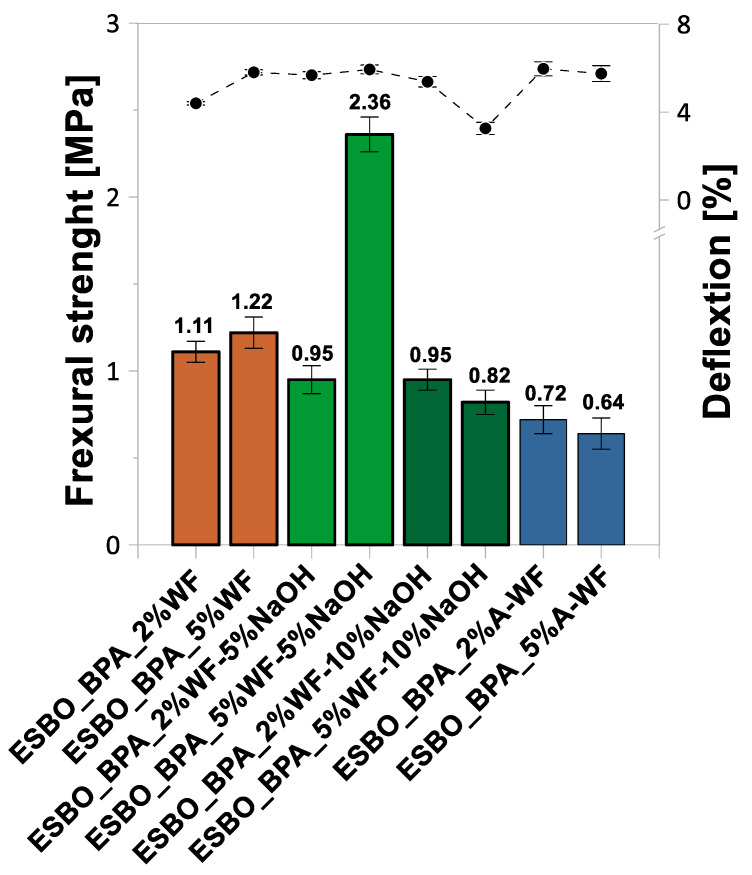
Flexural strength and deflection of compositions based on ESBO_BPA filled with waste oak flour.

**Figure 7:**
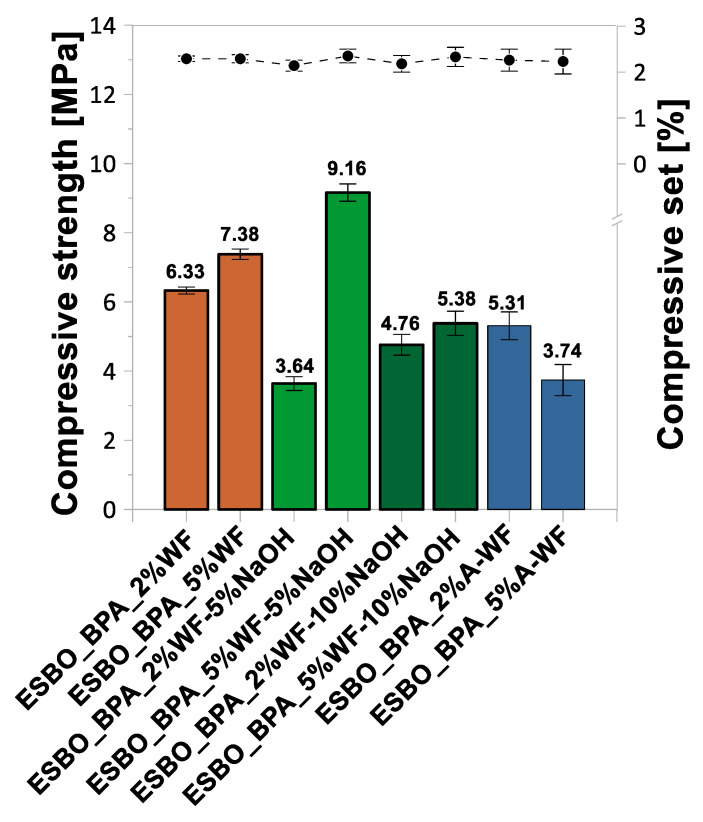
Compressive strength and compression set of compositions based on ESBO_BPA filled with waste oak flour.

Based on the research on the selected mechanical properties (Figure 5, Figure 6 and Figure 7, Table 2), such as the tensile strength, elongation at break, modulus elasticity, flexural strength, elasticity flexural modulus, deflection, compressive strength, compression set, hardness on the Shore A scale, and the impact strength without notches via the Charpy method, the influence of the wood waste modification on the characteristics of the obtained epoxy-polyurethane composites was analyzed. We found that the introduction of the unmodified wood waste into the composition resulted in the deterioration of the tensile, flexural, and compressive strengths compared to those of the samples without a natural filler (tensile strength—10.45 MPa, flexural strength—1.65 MPa, and compressive strength—11.27 MPa). The introduction of 2 wt % UM-WF resulted in a tensile strength of 6.95 MPa, while a 5% weight fraction of wood flour resulted in 6.75 MPa (Figure 5). An inverse relationship was observed for the modulus of elasticity; along with the increase in the weight share of the natural filler, its value increased from 129.13 MPa to 133.12 MPa. In addition, in the case of the flexural strength of the composites filled with UM-WF, lower values were recorded than those obtained for the material without wood filler (Figure 6). For the sample containing 2 wt % UM-WF, the value was 1.11 MPa; for the composition ESBO_BPA-5%UM-WF, the value was 1.22 MPa. Thus, an increase in the weight fraction of the WF in the tested composites resulted in an improvement in the flexural strength, while at the same time it reduced the value of the modulus of elasticity. Additionally, in the case of the compressive strength (Figure 7), the addition of UM-WF resulted in a decrease in the recorded values: from 11.37 MPa for the unfilled samples to 6.33 MPa for ESBO_BPA-2%UM-WF and 7.38 MPa for ESBO_BPA-2%UM-WF. Casado et al. [54] studied the properties of polyurethane composites based on tung oil and containing 10 and 15 wt % of wood flour. The obtained wood/polyurethane composites were characterized by a higher value for the tensile strength (by about 30% and 50%, respectively) than those registered for the unfilled polyurethane material. Additionally, Quirino et al. [55] reported on the preparation of soybean- and linseed oil-based thermosets reinforced with pine, oak, and maple wood flour. In general, it was found that increasing the filler loads led to an increase in the Young’s modulus and the tensile strength of the composites. Simultaneously, it was found that a comparison between the application of wood fiber and wood flour in polymeric composites indicated that samples reinforced with wood fibers were characterized by significantly higher mechanical properties. Moreover, after comparing the influence of the application of wood flour of different origins, it was stated that there were significant differences in the degree of reinforcement depending on the origin of the wood flour; among the tested wood particles, the best mechanical properties were obtained for pine wood flour. In turn, the application of chemically modified oak flour in compositions of polyaddition products based on modified soybean oil generally resulted in products characterized by similar or better mechanical properties than those obtained in the case of compositions filled with unmodified wood waste. The samples of ESBO_BPA_5%WF-5%NaOH were characterized by the highest tensile, flexural, and compressive strengths of all the obtained compositions based on the ESBO_BPA polyaddition product. In the case of samples containing 2 wt % of the wood filler modified with a 5% NaOH solution, relatively low tensile (5.72 MPa), flexural (0.95 MPa), and compression (3.64 MPa) strengths were obtained for the composition containing 5 wt % of WF-5%NaOH; the mechanical properties had improved significantly. The following values were recorded: tensile strength—10.86 MPa, flexural strength—2.36 MPa, and compression strength—9.16 MPa. However, for composites filled with wood flour mercerized using a 10% solution of NaOH, the opposite dependence was recorded. The increase in the weight fraction of the filler in the composition caused a deterioration of the mechanical properties. A sample containing 2 wt % of WF-10%NaOH was characterized by a lower tensile strength (6.64 MPa) compared to the one recorded for ESBO_BPA_5%WF-10%NaOH (8.29 MPa). In turn, the compressive strengths showed improved results for the composites containing 5 wt % of mercerized wood flour: 4.76 MPa for 2 wt % of WF-10%NaOH and 5.38 MPa for 5 wt % of the modified flour. The reason for obtaining better mechanical properties for compositions containing 5 wt % of mercerized oak flour could be the influence of the concentration of the used NaOH solution. In the case of the application of a solution with a lower concentration, the alkali treatment of the natural filler resulted in the purification of the material and the removal of lignocellulose and hemicelluloses from its structure, which caused the improvement in the adhesion between the filler and a polymer matrix [56]. On the other hand, an excessive concentration of alkali may adversely affect the surface of the natural filler. The use of a 10% solution of NaOH probably damaged the structure of the wood flour and hence reduced adhesion to ESBO_BPA. A similar relation was also pointed out by Gu et al. [57]. They showed an increase in the mechanical strength for polymer composites containing coconut fibers modified with NaOH solutions at a concentration in the range of 2–8%. However, in the case of higher concentrations, a significant deterioration of the analyzed properties was recorded. Therefore, we concluded that there was a certain limit of concentration versus the time of modification using the sodium hydroxide solution to improve the properties of composites with a natural filler modified by mercerization.

All materials based on the ESBO_BPA polyaddition product filled with the oak filler in various weight fractions were characterized by similar values of Shore A hardness (Table 2). Nevertheless, all recorded values for compositions containing wood flour were lower than those obtained for the reference sample. Among all compositions containing wood waste, the ESBO_BPA_5%WF-5%NaOH sample was characterized by the highest value of Shore A hardness (92.60°Sh), which equaled that of the sample without filler. Additionally, the compositions ESBO_BPA_5%UM-WF and ESBO_BPA_2%WF-10%NaOH were characterized by a higher impact strength (about 7 kJ/m^2^) than that of the pure ESBO_BPA sample (5.85 kJ/m^2^). 

### 3.3. Results of SEM Analysis of ESBO_BPA/Wood Flour Composites

**Figure 8:**
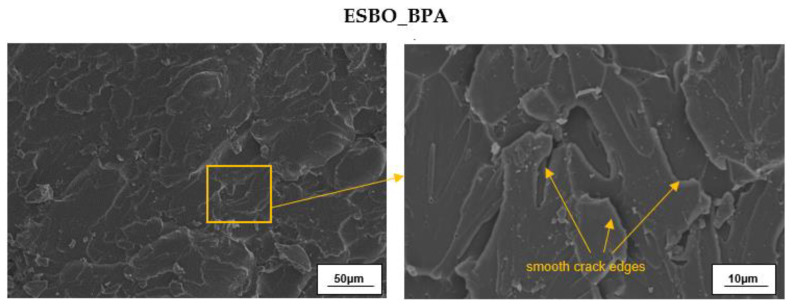
SEM micrographs of the impact fracture surface of the crosslinked sample of reference sample ESBO_BPA.

Based on the SEM analysis (Figure 8) performed on the surfaces of the impact fractures of pure cross-linked epoxy-polyurethane compositions, we found that the cured ESBO_BPA material was characterized by a homogeneous structure without any phase separation, which is typical for samples of cross-linked epoxy materials with the addition of vegetable oils as reactive diluents [58]. 

**Figure 9:**
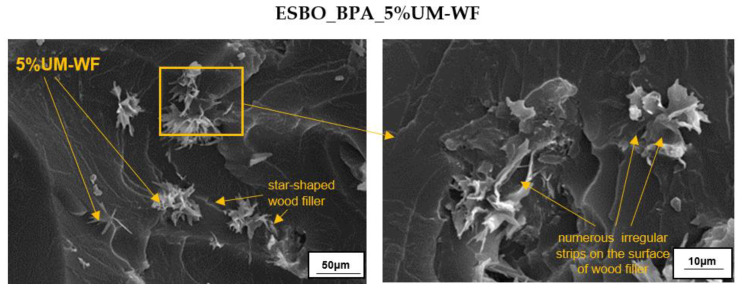
SEM micrographs of the impact fracture surface of the crosslinked sample of ESBO_BPA composition filled with 5 wt % of unmodified wood flour.

In the case of such materials, oil microdomains that were evenly dispersed throughout the sample were observed. Theoretically, in the case of epoxy–polyurethane materials, which were the subject of the present research, the phenomenon of phase separation may occur, due to both the different polarity of the fusion product macromolecules and bisphenol A as well as the different reactivity of the secondary hydroxyl and phenolic groups. However, as indicated above, based on the performed SEM analysis, no such phase separation in the case of the pure ESBO_BPA was observed. Additionally, it is worth mentioning that the obtained epoxy–polyurethane compositions also did not show brittle cracks, which are typical for epoxy materials based on petrochemical raw materials. In turn, the SEM microphotographs of biocomposites filled with the unmodified oak filler (Figure 9) revealed agglomerates of the biofiller that were unevenly distributed throughout the composition. Simultaneously, the biofiller particles were characterized by an interesting star shape. The agglomeration of the biofiller might be explained by its poor interfacial affinity, resulting in the lower values for the mechanical properties recorded for the ESBO_BPA-UM-WF samples. 

**Figure 10:**
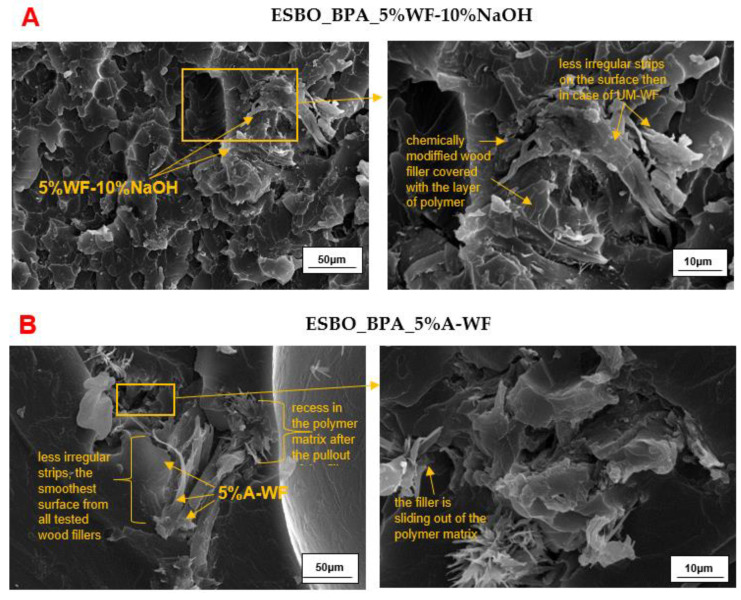
SEM micrographs of the impact fracture surfaces of the crosslinked samples of (**A**) ESBO_BPA composition filled with 5 wt % of wood flour mercerized using a 10% solution of NaOH and (**B**) ESBO_BPA composition filled with 5 wt % of acetylated wood flour.

Unfortunately, the performed chemical modification only slightly led to a better distribution of the biofiller (Figure 10A,B). However, the interface between individuals—the polymeric matrix and the modified oak flour—was still noticeable in the form of a fiber pullout from the polymer matrix. On the other hand, we found that the biofiller was more coated by the polymer material than in the case of the samples with UM-WF. Additionally, what we observed in the microphotographs of samples containing the modified biofiller could undoubtedly be linked to the results of the performed mechanical tests. As indicated above, among all tested compositions, the samples containing 5 wt % of WF-5%NaOH were characterized by the best mechanical properties, which resulted from the most effective interference of the biofiller and the polymeric matrix. Such interaction of the components was possible due to the most effective removal of not only the residues of various impurities, but also of lignocellulose and hemicelluloses from the structure of the oak filler. 

## 4. Conclusions

The aim of undertaken research was to develop a method for managing wood-processing waste, which, unfortunately, mainly ends up in landfills, except for minor use in filling wooden floors. The parquet flooring post-production wood flour was applied as reinforcement for epoxy resins based on a polyaddition product of epoxidized soybean oil and bisphenol-A. As presented within this manuscript, previous studies belonging to a wide variety of application-related research focused on the utilization of green fillers such as post-agricultural waste powder, grass fibers, bast/leaf fibers, and other natural fibers as reinforcement for both synthetic and bio-based epoxy resins [42,59,60]. The search for the possibility of additional uses continues because the management of waste or post-production particles of natural origin is environmentally friendly due to their sustainability, low cost, and wide availability. Both unmodified and chemically modified oak wood waste was used in our study. We subjected the biofiller to the chemical modification in order to obtain a reduction in the hydrophilicity of the wood. The performed mercerization and alkalization affected the reduction in the content of the hydroxyl groups and various impurities of the biofiller and usually resulted in better polymer matrix/filler adhesion. We noted that acetylation of the oak wood waste led to a better elimination of the hydroxyl groups compared with mercerization. However, as recorded in the SEM microphotographs, acetylation also led to the removal of irregular strips from the surface of the bio-filler and smoothing of its surface, resulting in a poorer surface wettability by the polymer and consequently an easier pullout of the filler from the polymer matrix. This in turn resulted in registering the worst mechanical properties of all of the analyzed samples. The best properties were achieved for the cured samples of ESBO_BPA filled with 5 wt % of oak flour that was mercerized using a 5% solution of NaOH. At the same time, we found that the concentration of NaOH at a level of 10% deteriorated the mechanical-strengthening properties of the wood filler. All of the obtained epoxy biocomposites contained about 60% raw materials of natural origin.

## Figures and Tables

**Table 1:** FT-IR analysis of unmodified and modified waste oak flour [51,52,53].

Frequency (cm^−1^)					Associated Band		
UM-WF	WF-5%NaOH	WF-10%NaOH	A-WF	D-WF			
3607–3080	3560–3080	3600–3035	-	3642–2999	*vb*	-OH	*v* valencene
2980–2822	2965–2785	2996–2785	2938	2992–2812	*w*	-CH_3_	*v* valencene
-	-	-	-	2271	*s, asym*	-N=C=O	*v* valencene
1728.89	-	-	1735	1725	*s*	-C=O	*v* valencene
1595.81	1587.13	1590.02	1593	-	*vb*	-C=C-	skeletal ring
-	-	-	-	1365	*s-m, sym*	-CH	*δ* deformation
1297–1188	-	-	1286–1180	1278–1194	*w*	-C-O	*v* valencene
1178–860	1172-883	1172-930	1140–942	1167–915	*sym*	-C-O	*v* valencene
-	-	-	600	-	*s*	-COCO	*δ* deformation

*Intensity: w*—weak; *s*—strong; *m*—medium; *vb*—variable; *v*—tensile vibrations; *δ*—deformation; *sym*—symmetrical; *asym*—asymmetrical.

**Table 2:** Mechanical properties of compositions based on ESBO_BPA filled with wood flour.

Mechanical Properties	Tested Epoxy–Polyurethane Compositions Based on ESBO_BPA							
	Unmodified Wood Flour		Modified Wood Flour					
			5% NaOH		10% NaOH		Acetylation	
	2%WF	5%WF	2%WF	5%WF	2%WF	5%WF	2%WF	5%WF
Modulus of elasticity (MPa)	129.13 ± 17.31	133.12 ± 18.40	117.07 ± 11.01	236.92 ± 30.15	144.85 ± 11.29	215.30 ± 29.64	132.33 ± 9.98	80.24 ± 4.86
Elasticity flexural modulus (MPa)	163.33 ± 31.21	115.20 ± 9.83	106.75 ± 9.71	229.20 ± 19.63	121.25 ± 24.60	124.00 ± 16.09	64.00 ± 24.00	81.67 ± 16.65
Shore Hardness (Sh°A)	90.2 ± 4.2	88.5 ± 2.7	92.4 ± 2.6	92.6 ± 3.1	90.9 ± 4.1	91.1 ± 2.1	87.5 ± 2.5	90.4 ± 2.9
Impact toughness (kJ/m^2^)	4.64 ± 0.57	7.00 ± 1.89	3.24 ± 0.22	4.52 ± 0.25	6.99 ± 0.89	3.90 ± 0.85	4.88 ± 1.82	4.24 ± 0.65

## Data Availability

Data available on request.
